# 

**Published:** 1850-02

**Authors:** 


### NOTICE TO CORRESPONDENTS.

Communications and Books for notice should be addressed to the Editor, care of Messrs. Lindsay & Blakiston.

Letters, &c., connected with the *business affairs* of the Journal should be addressed to the Publishers.

Papers for publication must be received *before* the 20th of the month, or they cannot appear in the forthcoming number.

The following Journals and Publications have been received since Jan. 1st:

The Boston Medical and Surgical Journal. (Weekly, Boston.)
Buffalo Medical Journal.
Western Journal of Medicine and Surgery.
Medical News.
Western Lancet.
American Journal of Insanity.
The American Journal of Medical Sciences.
New Orleans Medical and Surgical Journal.
Ohio Medical and Surgical Journal.
Charleston Medical Journal and Review.
Transactions of the American Medical Association.
Southern Medical and Surgical Journal.
The British American Journal of Medical and Physical Science. (Montreal.)
The London Lancet. (Weekly, London.)
The Medical Times. (Weekly, London.)
Journal of Psychological Medicine. Oct., Nov. and Jan.
Dublin Quarterly Journal. Nov.
London Journal of Medicine. Nov., Dec. and Jan.
British and Foreign Medlco-Chirurgical Review. Jan. 1850.
London Medical Gazette. Dec. and Jan.
Provincial Medical and Surgical Journal. Dec. and Jan.
Dublin Medical Press. Dec.
Braithwaite's Retrospect.
Révue Medico Chirurgicale de Paris. Novembre.
Pharmaceutical Journal. Nov., Dec. and Jan.
Carnoghan on Congenital Dislocations of the Femur. (From the Publishers.)
Prof. Howard's Introductory Lecture.
Prof. J. K. Mitchell's " "
Prof. P. F. Eve's " "
Prof. J. F. Sanford's " "
Prof. W. Gibsons " "
Circular of Geneva College.

---

Communications have been received from:—
R. G. Meredith, M. D., Canterbury, Va.
J. T. Hearne, M. D., Lowndsboro, Ala.
T. E. Waller, M. D., Camden, Del.
E. C. Banks, M. D., Lawrenceville, Ill.
D. B. Hilliard, M. D., Halifax Co., N. C.
John Smith, M. D., Kingston, Pa.
Wm. H. Gobrecht, M. D., Philadelphia.
Jas. Bryan, M. D., Philadelphia.

---

The foreign correspondents of the Examiner will please direct their Exchanges and other communications to the care of Mr. Charles J. Skeet, 27 King William St., Charing Cross, London, or Mr. H. Bossange, 21 Bis, Quai Voltaire, Paris.

MEDICAL SOCIETY OF THE STATE OF PENNSYLVANIA.

The second annual meeting of this Society will be held in Philadelphia on the third Wednesday in April, (17th.)

